# The role of Optical Coherence Tomography in optic neuropathies

**Published:** 2018

**Authors:** Raluca Eugenia Iorga, Andreea Moraru, Manuela Ramona Ozturk, Dănuţ Costin

**Affiliations:** * Department of Ophthalmology, “N. Oblu” Clinical Emergency Hospital, Iaşi, Romania; ** Department of Ophthalmology, “Gr. T. Popa” University of Medicine, Iaşi, Romania

**Keywords:** optical coherence tomography OCT, optic neuropathy, multiple sclerosis MS, retinal nerve fiber layer RNFL, ganglion cells layer GCL

## Abstract

Optical neuropathies are neuro-ophthalmologic disorders, the main symptoms of which are the decrease of visual acuity and the alteration of the color vision. Optical coherence tomography has been one of the most important innovations in ophthalmology, which offered the possibility to analyze specific structures of the retina. Optical coherence tomography performs in vivo, real-time, noncontact scanning and provides cross-sectional and volumetric images with a resolution approaching that of histology. Optical coherence tomography offers the opportunity to study neurological diseases in an objective and non-invasive manner. The measurements of retinal nerve fiber layer can be an objective measurement of nerve swelling or nerve atrophy. By analyzing the ganglion cell complex, optical coherence tomography can help detect early axonal damage and may predict the visual outcome. It can be useful for diagnosis and follow-up of optic nerve and chiasmal compressive diseases. Furthermore, optical coherence tomography is useful in patients with multiple sclerosis in distinguishing macular disease from optic neuritis and in monitoring the treatment. Multiple studies and clinical observations support the importance of optical coherence tomography in the diagnosis, treatment, and follow-up of optic neuropathies.

Abbreviations: OCT = optical coherence tomography, VA = visual acuity, RNFL = retinal nerve fiber layer, GCL = ganglion cells layer, MS = multiple sclerosis, ON = optic neuropathy, NAION = non-arteritic ischemic anterior optic neuropathy, LHON = Leber hereditary optic neuropathy, RE = right eye, LE = left eye

## Introduction

Optic neuropathy refers to damage to the optic nerve due to multiple causes. Damage and death of these neurons leads to characteristic features of optic neuropathy. The main symptoms are a decrease of visual acuity (VA) and the alteration of the color vision, with colors appearing subtly washed out in the affected eye. On clinical examination, the optic nerve head appears edematous in early stages. A pale disc is characteristic of long-standing optic neuropathy. Optic atrophy is the end result of any disease that damages nerve cells anywhere between the retinal ganglion cells and the lateral geniculate body. 

Optical Coherence Tomography (OCT) is a non-invasive method that makes the introduction to high-tech medicine, a technique that revolutionizes the early detection of ocular and central nervous system disorders (Alzheimer’s, Parkinson’s, multiple sclerosis or vascular dementia). OCT is a method capable of producing two-dimensional cross-sections of the retina with very good spatial resolution. OCT has a wide use in ophthalmology, especially in the diagnosis and monitoring of a wide variety of retinal diseases affecting the macula, as well as calculates the thickness of the retina [**1**]. The operation of the OCT is based on an optical measurement technique called “low coherence interference”. When light emitted by the source of the device is directed to the eye, it is reflected by intraocular structures with different optical properties. The OCT uses coherent laser light to sweep the retina and analyze the light reflected by the retinal layers. This way, an in vivo “biopsy” of the retina provides information of high quality resolution about all its layers [**2**,**3**]. The OCT allows qualitative (localization, shape, structure) and quantitative analyzes (retinal measurements, especially of the retinal thickness and retinal nerve fiber layer RNFL) [**4**].

Retinal layers:

1. Retinal pigment epithelium layer

2. Layer of the external segment of photoreceptor cells

3. External limiting membrane

4. The external nuclear layer

5. External plexiform layer

6. The internal nuclear layer

7. Internal plexiform layer

8. Ganglion cells layer (GCL)

9. Nerve fibers layer

10. Internal limiting membrane

**OCT in different optic neuropathies**

**1. Optic neuritis in multiple sclerosis**

Optic neuropathy (ON) may be the first clinical demyelinating event in up to 20% of the patients with multiple sclerosis (MS) and the probability of developing clinically-definite MS was 50% by 15 years after the onset of acute ON [**5**]. While visual recovery from ON is considered as a first demyelinating event [**6**], studies showed that patients would have deficits that are not well captured by high-contrast visual acuity alone [**7**]. 

The OCT exam allows a correlation between the structural aspect (neuronal loss) and visual dysfunction. The OCT exam shows a thinning of the RNFL and the ganglion cells layer GCL, and can be used as a marker in the follow up of multiple sclerosis patients. Trip et al. reported a 33% reduction in peripapillary RNFL thickness in eyes with a history of ON and incomplete recovery. There was a 27% reduction in the affected eyes compared to the unaffected fellow eyes [**8**]. The OCT measurements showed both axonal loss and retinal ganglion cells loss.

**Fig. 1:**
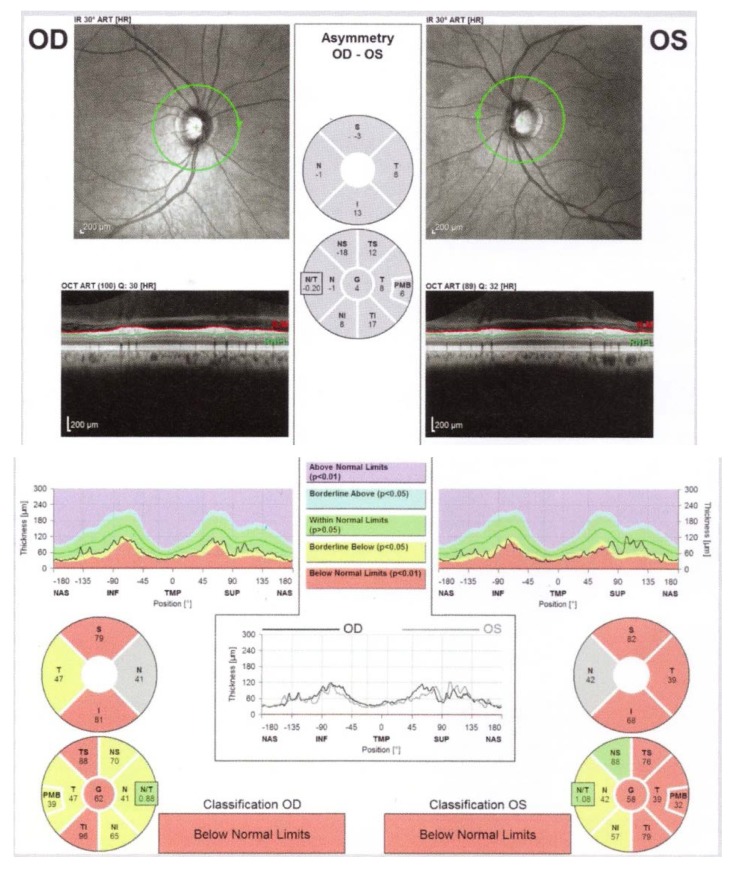
Bilateral RNFL thinning in MS patient with no optic neuritis (from Andrzej Grzybowski, Piero Barboni, OCT in central nervous system disease. The eye as a window to the brain, 2016, Switzerland, Springer International Publishing, ISBN 978-3-319-24083-1, pg 97)

The decrease in the peripapillary RNFL thickness by approximately 10-40μ is maximal at 3-6 months after the acute episode, and a stabilization is observed at 7-12 months [**9**] (**Fig. 1**).

OCT was able to identify mild and even clinically undetectable optic disc edema in eyes with acute ON [**10**]. In 2006, Fisher et al. compared the RNFL thickness among MS eyes with a history of ON, MS eyes without a history of ON and disease-free eyes. The RNFL thickness was reduced significantly among MS eyes as a group overall (92 μ) vs. controls (105 μ) and particularly reduced in MS ON eyes (85 μ) [**11**].

**Fig. 2:**
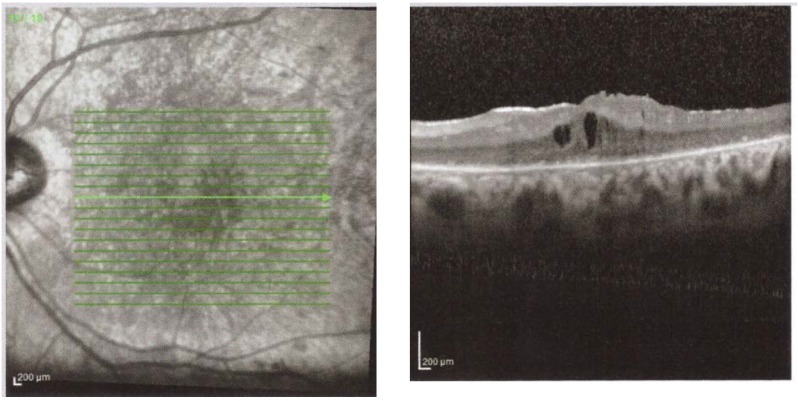
Fingolimod related macular edema (from Andrzej Grzybowski, Piero Barboni, OCT in central nervous system disease. The eye as a window to the brain, 2016, Switzerland, Springer International Publishing, ISBN 978-3-319-24083-1, pg 98)

OCT plays a role in monitoring the treatment with fingolimod and INF-β. Fingolimod can cause macular edema (0.3-1.2% of the patients) and uveitis [**12**], while INF-β can cause exudates and retinal hemorrhage [**13**]. An OCT exam is recommended before treatment, and one at every 3-4 months (**Fig. 2**).

Patients with DeVic disease can present microcysts in the internal nuclear layer on OCT - signs of inflammation, autoantibodies against aquaporin 4, microglial activation, neurodegenerative changes [**14**].

**2. Non-arteritic ischemic anterior optic neuropathy**

Non-arteritic ischemic anterior optic neuropathy NAION is characterized by abrupt decrease of VA, associated with papillary edema and peripapillary hemorrhage. NAION is secondary to the reduction of blood flow to the head of the optic nerve by the phenomenon of crowding on a small excavation disc. 

**Fig. 3:**
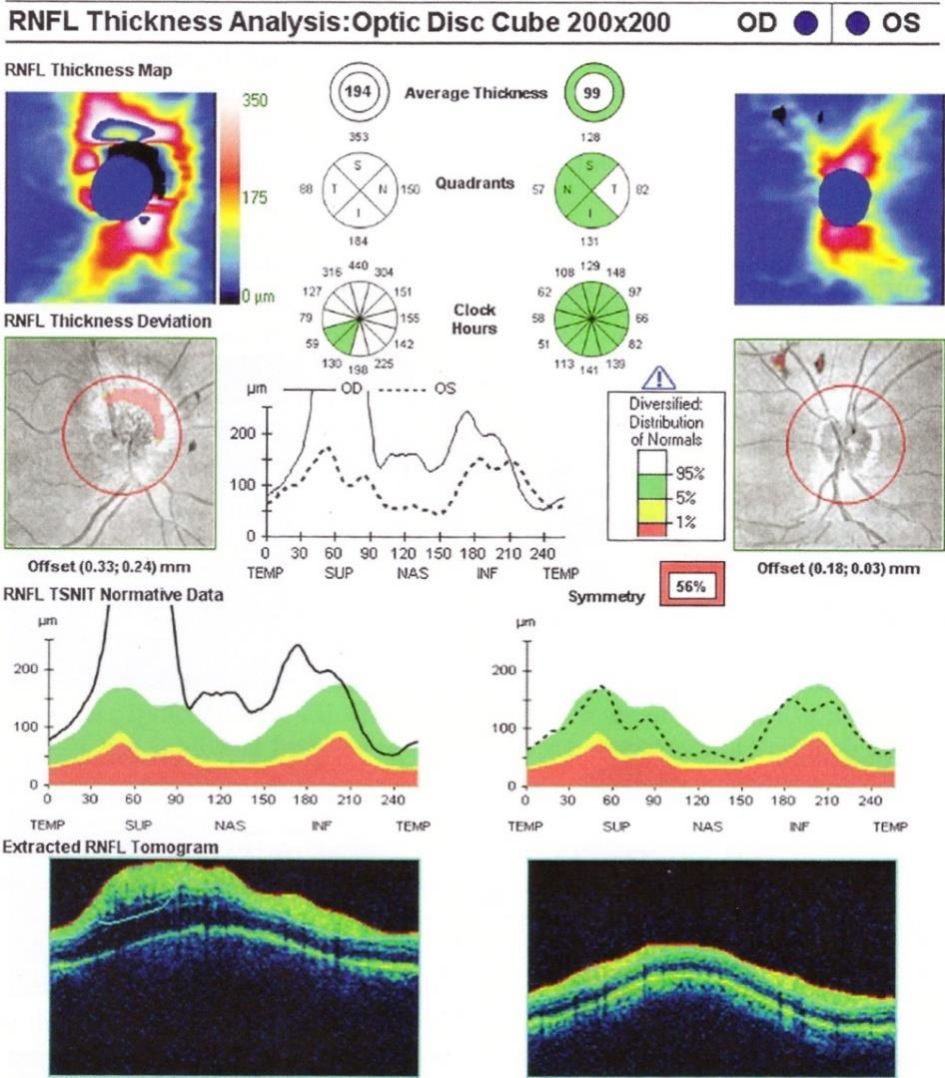
Right Eye RE Optic disc edema secondary to NAION (from Andrzej Grzybowski, Piero Barboni, OCT in central nervous system disease. The eye as a window to the brain, 2016, Switzerland, Springer International Publishing, ISBN 978-3-319-24083-1, pg 54)

At presentation, the OCT shows the RNFL edema. Histopathological studies have shown a doubling of RNFL in NAION [**15**]. In time, the edema reduces, and we can observe a thinning of the RNFL corresponding to optic nerve atrophy, on OCT. At 2 months post acute episode, the RNFL thickness in the affected eye is similar to that of the congenital eye and at 3-4 months, there is a 40% decrease in the thickness of the RNFL compared to the healthy eye. At 12 months, the RNFL thickness stabilizes [**16**,**17**] (**Fig. 3**).

**Fig. 4:**
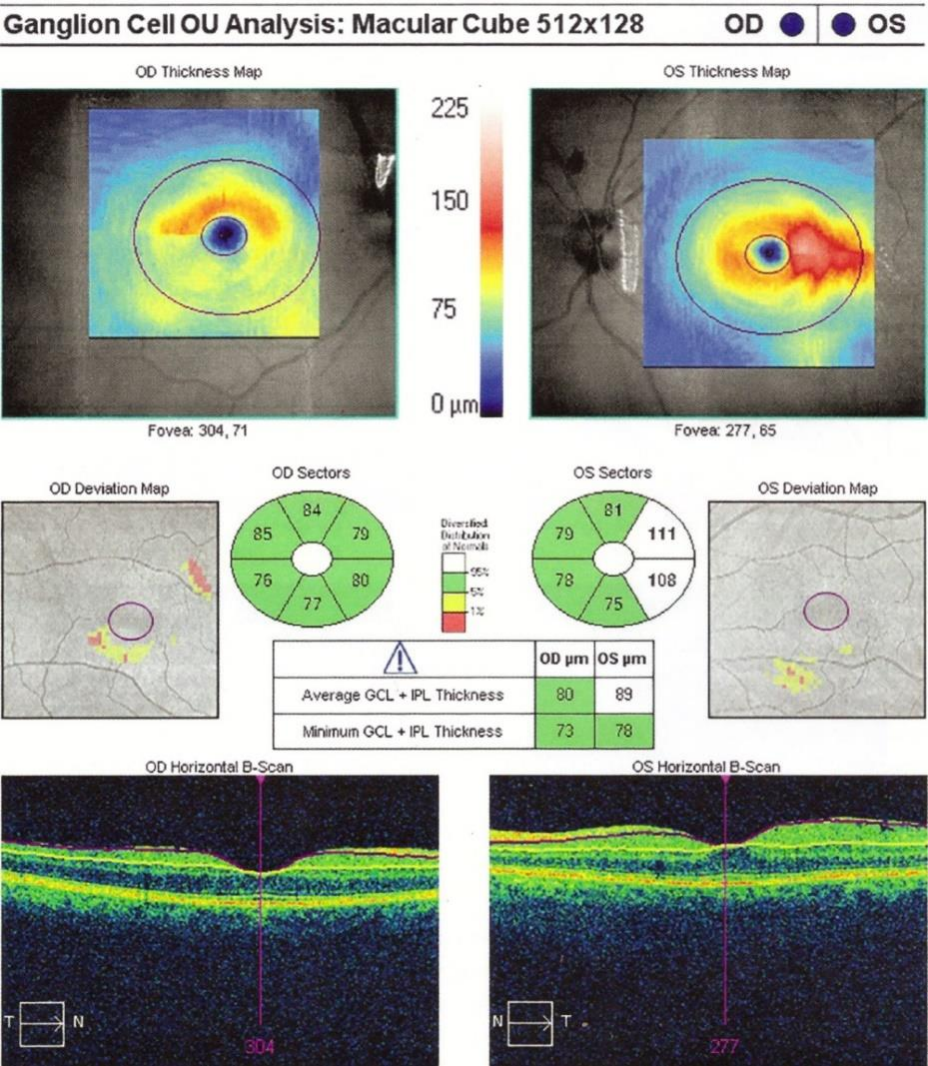
RE Thinning of the GCL in a patient with NAIO (from Andrzej Grzybowski, Piero Barboni, OCT in central nervous system disease. The eye as a window to the brain, 2016, Switzerland, Springer International Publishing, ISBN 978-3-319-24083-1, pg 53)

Certain patients with secondary papillary edema NAION develop subretinal fluid. OCT shows peripapillary subretinal hyporeflectivity adjacent to RNFL thickening and subfoveolar hyporeflectivity [**18**]. Hedges observed that 8 out of 76 eyes with NAION had subfoveal fluid, at 4 weeks after the acute phase [**18**]. In NAION, it appears to be a more significant damage to the maculopapillary fibers, which corresponds to a central defect. Papchenko found a strong correlation between macular thickness and visual field sensitivity. Macular thickness may be a surrogate marker for determining the extent of optic nerve damage [**19**]. Ganglion cells thickness correlates with visual field parameters, both in acute and chronic phase. Ganglion cells thickness measured by OCT can detect axonal damage when the RNFL is edematous [**20**] (**Fig. 4**).

**3. Leber hereditary optic neuropathy (LHON)**

Leber hereditary optic neuropathy is an optic neuropathy associated with mutations in mitochondrial DNA that affects young men. It is associated with retinal ganglion cell degeneration and axonal loss in the optic nerve, leading to optic atrophy.

In healthy carriers, the OCT test showed RNFL thickening in temporal quadrants and in asymptomatic men in the inferior sector [**21**]. RNFL thickening is correlated with axonal edema, with mitochondrial redistribution at dysfunctional ganglion cells, affecting the prelaminar unmyelinated portion of the optic nerve axons [**22**].

**Fig. 5:**
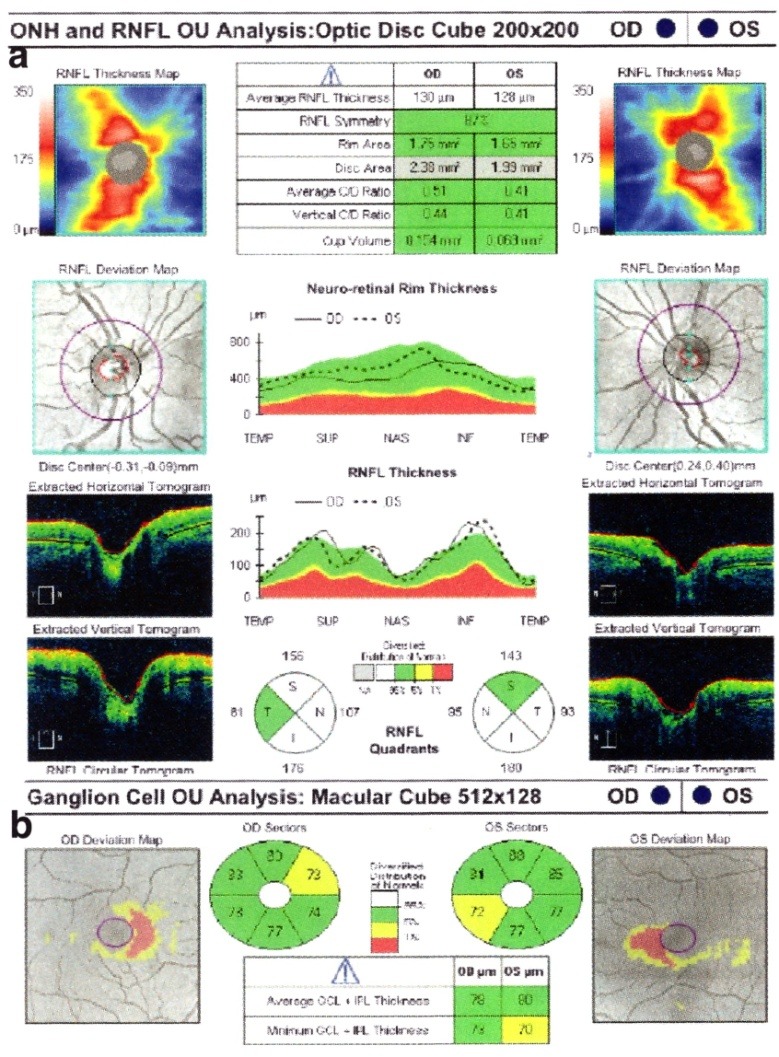
Acute phase of LHON with a. peripapillary RNFL thickening and b. early GCL atrophy (from Andrzej Grzybowski, Piero Barboni, OCT in central nervous system disease. The eye as a window to the brain, 2016, Switzerland, Springer International Publishing, ISBN 978-3-319-24083-1, pg 188)

**Fig. 6:**
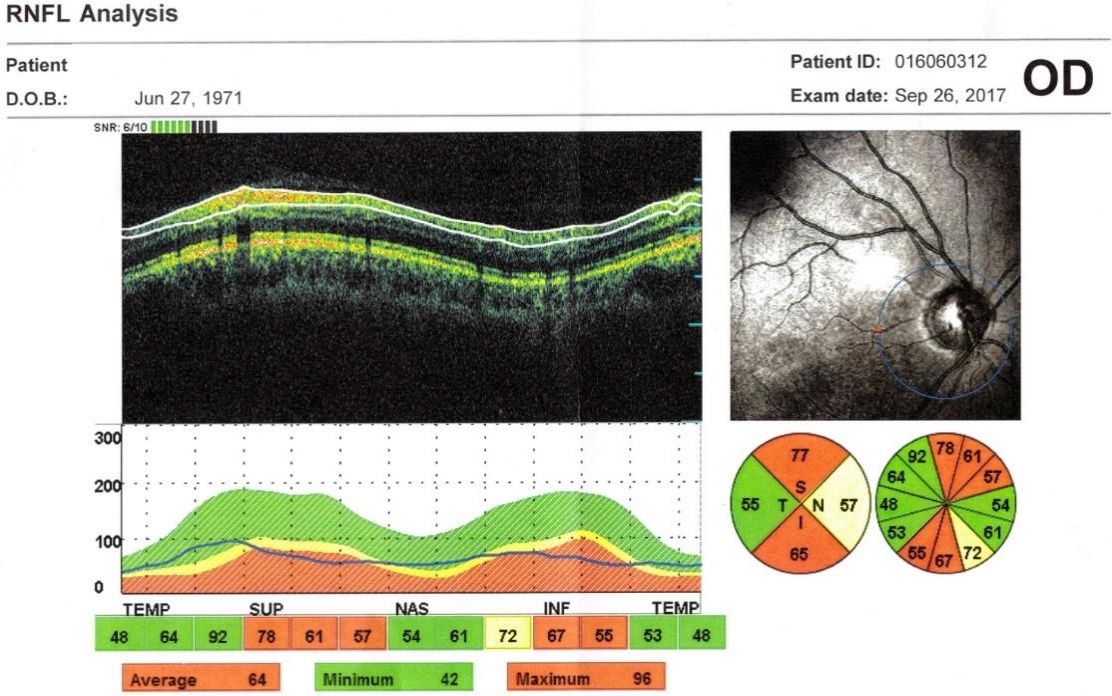
RNFL thinning in atrophy phase in a patient with LHON

In the acute phase and in the first 6 months of onset, the RNFL analysis shows thickening in the upper and lower segments, compared to the control group [**23**] (**Fig. 5**). At the atrophic stage, a RNFL thinning appears in all segments (**Fig. 6**). Patients who recover from VA have a higher RNFL thickness than those who do not recover [**23**]. 

The OCT analysis of macular thickness showed a thinning of the GCL at the early stage in the sectors of the inner ring [**24**]. 6% of the patients had microcystic degeneration and were associated with focal retinoschisis induced by vitreomacular tractions in combination with RNFL atrophy [**25**]. 

**4. Compressive optical neuropathies**

Compressive optical neuropathies are among the most important anterior optical pathways diseases that can lead to severe impairment of visual function if they are discovered late. The diagnosis is made through a progressive decline of VA associated with imaging exams. Axonal loss can be quantified by peripapillary RNFL measurements, as well as by measuring the macular thickness. OCT is useful in the diagnosis, follow-up, and visual prognosis of patients with compressive optic neuropathies. Among the causes, the intrinsic causes are listed - glioma, optic nerve meningioma, and extrinsic, compressive causes - orbital tumor, Graves disease, intracranial tumors (pituitary adenoma, craniopharyngioma), aneurysm.

**Fig. 7:**
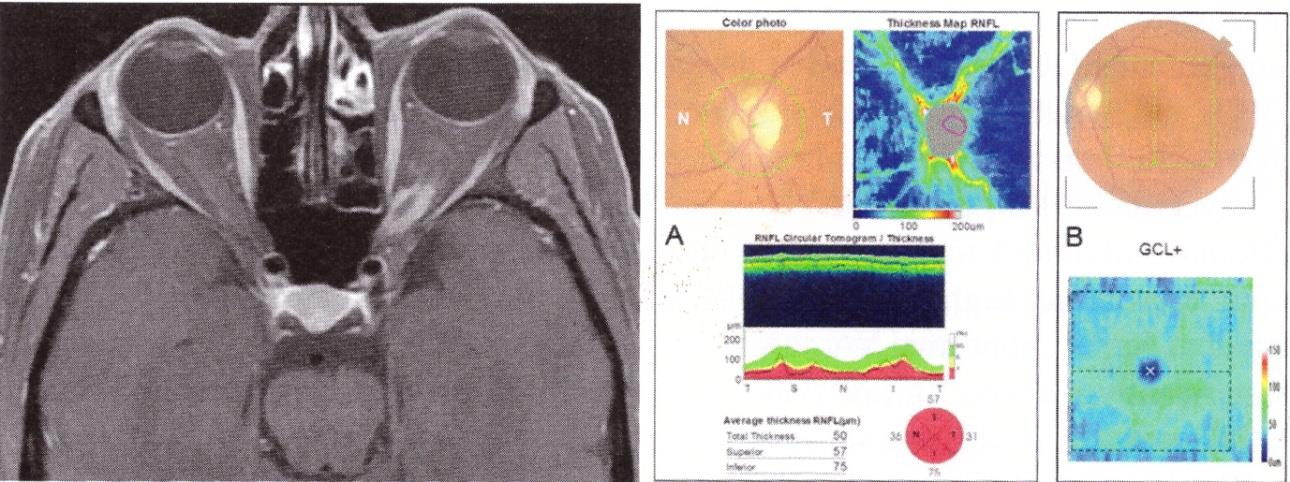
Left Eye LE optic nerve meningioma IRM and OCT. A. Thinning of the RNFL on OCT. B. Reduced GCL on OCT (from Andrzej Grzybowski, Piero Barboni, OCT in central nervous system disease. The eye as a window to the brain, 2016, Switzerland, Springer International Publishing, ISBN 978-3-319-24083-1, pg 73)

Meningioma occurs with the progressive decrease of VA. The OCT exam is important in predicting the visual prognosis: when the VA decrease occurs in a patient with minimal RNFL loss, the prognosis is favorable. Loo evaluated 14 eyes of 12 patients with pretreatment and post-treatment OCT, along with clinical examination. After treatment, the group with normal RNFL experienced improvement in VA, color vision, visual field, compared with the group with altered RNFL [**26**] (**Fig. 7**). 

Glioma - Avery et al. studied 62 patients with NF-1 and optic nerve glioma. OCT showed a reduction in RNFL thickness, which significantly associates with VA decrease and sensitivity to contrast [**27**]. Fard et al. included 38 eyes from 23 children with optic nerve glioma and they correlated the OCT exam with clinical and radiological changes. The RNFL and macular OCT measurements were significantly different in patients with tumor progression [**28**]. 

**Fig. 8:**
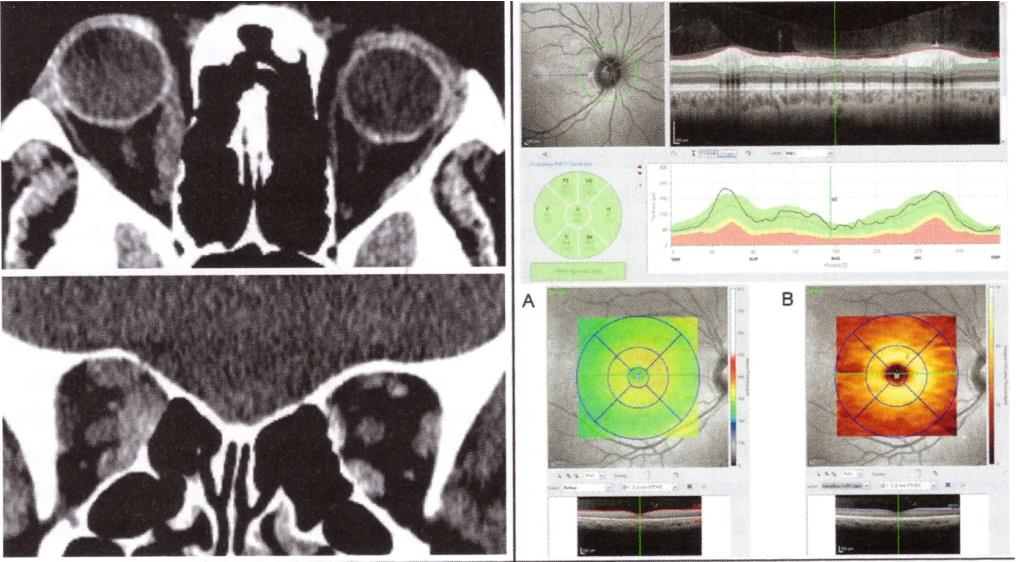
Patient with Graves’ orbitopathy - IRM and OCT. IRM shows enlarged medial and inferior rectus muscles. OCT shows normal peripapillary RNFL, normal macular full-thickness (A) and normal GCL (B). (from Andrzej Grzybowski, Piero Barboni, OCT in central nervous system disease. The eye as a window to the brain, 2016, Switzerland, Springer International Publishing, ISBN 978-3-319-24083-1, pg 76)

Graves Orbitopathy is an autoimmune inflammatory disorder that affects extraocular muscles and orbital fat, which are responsible for proptosis, diplopia, congestive signs, and dysthyroid optic neuropathy. Because prognosis is significantly improved when early diagnosis is made, OCT can be used to detect high-risk patients. The OCT measurements of the RNFL can predict axonal losses. Forte et al. observed RNFL losses in 30% of the patients with dysthyroid optic neuropathy and intraocular hypertension [**29**]. After decompression, an improvement in VA and visual field in patients with normal retinal integrity was marked, as assessed by OCT [**29**] (**Fig. 8**).

**Fig. 9:**
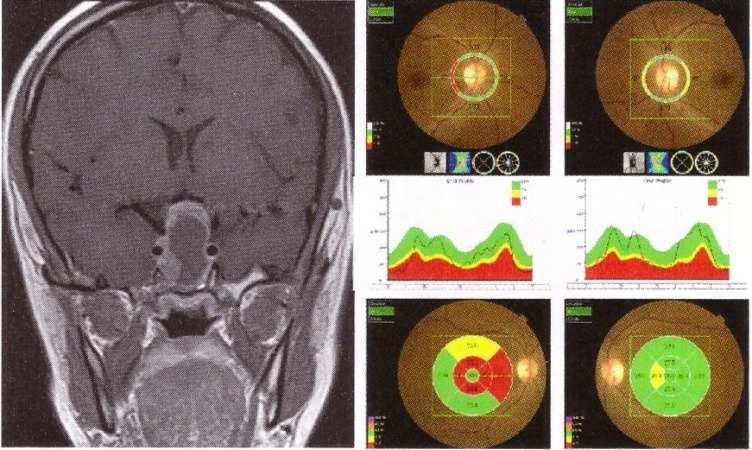
IRM and OCT of a patient with large pituitary adenoma with chiasmal compression. OCT shows reduced peripapillary RNFL - temporal in the RE and nasal and temporal in the LE (upper image) and thinning of the nasal macula (lower image). (from Andrzej Grzybowski, Piero Barboni, OCT in central nervous system disease. The eye as a window to the brain, 2016, Switzerland, Springer International Publishing, ISBN 978-3-319-24083-1, pg 82)

Chiasmal lesions may be caused by pituitary adenoma, craniopharyngioma, meningioma, and aneurysm. Chiasmatic lesions can cause changes ranging from minimal to severe, with optic nerve atrophy and ganglion cells loss. The OCT measurements of retinal thickness can detect neuronal loss from onset. Patients with bitemporal hemianopsia show RNFL losses in all optic nerve quadrants and a macular thinning nasal from the fovea [**30**] (**Fig. 9**). Danesh-Meyer evaluated 40 patients with chiasmatic lesions, with OCT and visual field, pre and post decompression treatment. Patients with thin RNFL did not demonstrate significant improvement in VA and visual field as compared to those with normal RNFL [**31**].

Using OCT to quantify axonal losses by measuring RNFL and macular thickness can be considered a predictor for vision recovery. Jacob et al. showed that the increase in RNFL thickness correlated with visual field improvement [**32**].

**5. Nutritional and Toxic Optic Neuropathies**

Nutritional Optic Neuropathy is common in patients with chronic diseases and malnutrition, being associated with deficiency of B1, B12, folic acid, Cu. Deficiency in these elements affects mitochondrial oxidative phosphorylation [**33**]. The OCT measurements show a thickening of the RNFL, followed by a more pronounced thinning in the temporal sector [**34**].

Toxic Optic Neuropathies occur due to accidental ingestion of substances such as alcohol (methanol, ethylene glycol), anti-tuberculosis drugs, antimalarial drugs, antiarrhythmic (digital, amiodarone), and antibiotics. They present with painless, progressive, bilateral visual acuity decrease, dyschromatopsia, and central scotoma. The site can be variable, including the retina, intraocular nerve fiber axons, and chiasm. The symptomatology is due to the damage of the papillomacular bundle, which is more susceptible due to the large unmyelinated segment and thin gauge [**35**].

In early stages, toxicity accumulates in the mitochondria of the ganglion cells, causing axonal thickening, which is detectable in OCT. In the subacute stage, the OCT shows RNFL thinning of the papillomacular bundle in the inferior-temporal sector. In the late stage, RNFL thinning is observed in all layers [**35**,**36**].

Toxic optic neuropathy to ethambutol may become clinically manifested between 2 and 12 months after the onset of treatment. It is reversible if it is recognized on time. Permanent toxic effects may occur at standard doses of 15-25 mg/ kg/ day. Early animal experiments have demonstrated ethambutol toxicity affecting the retinal ganglion cells, the optic nerve, chiasm, and optic tracts [**37**]. In the late-stage, the OCT changes are the thinning of the RNFL in the inferior-temporal sector and significant thinning of the ganglion cells layer [**38**]. 

**Fig. 10:**
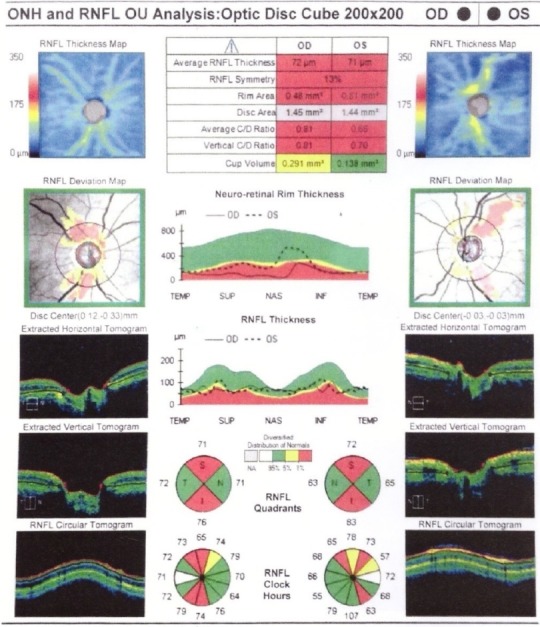
Methanol optic neuropathy - OCT shows thinning of peripapillary RNFL in both eyes (from Andrzej Grzybowski, Piero Barboni, OCT in central nervous system disease. The eye as a window to the brain, 2016, Switzerland, Springer International Publishing, ISBN 978-3-319-24083-1, pg 221)

**Fig. 11:**
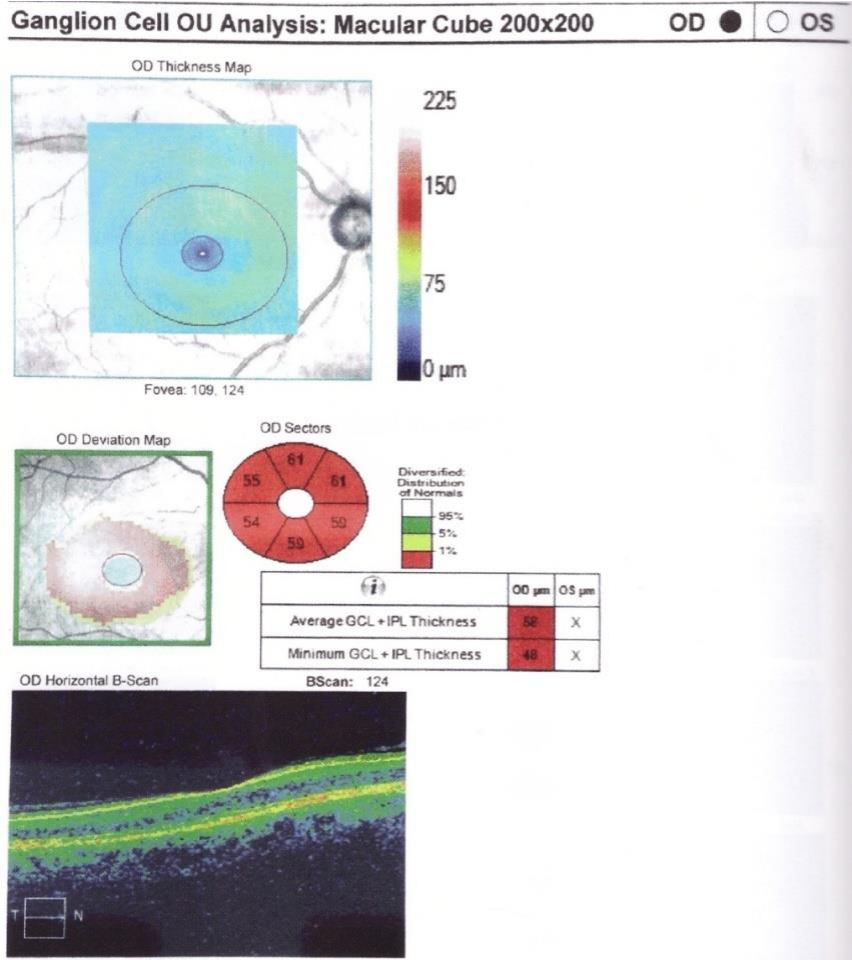
Methanol optic neuropathy - OCT shows decreased volume of GCL (from Andrzej Grzybowski, Piero Barboni, OCT in central nervous system disease. The eye as a window to the brain, 2016, Switzerland, Springer International Publishing, ISBN 978-3-319-24083-1, pg 222)

Methanol optic neuropathy is due to a cytotoxic edema at the retinal level and at the optic nerve, which appears at 48h post ingestion. OCT is used for the exploration of the macula and peripapillary retina, showing early peripapillary RNFL edema, subsequent optic nerve atrophy, and intraretinal fluid accumulation [**39**] (**Fig. 10**,**11**).

Toxic optic neuropathy to amiodarone can be confused with NAION. It has an insidious onset with a less severe VA decrease and optic nerve edema. As a mechanism, we note the accumulation of cytoplasmic inclusions in lysosomes. OCT changes show RNFL thickening in early stages, followed by axonal loss and optical atrophy [**40**].

Tobacco toxic optic neuropathy progressively affects the papillomacular bundle, which correlates to central and cecocentral scotomas. In early stages, the optic nerve appears normal, with hemorrhages and dilated vessels [**41**].

## Conclusions

OCT is a major step forward in ophthalmology, allowing the obtaining of in vivo images of the retina and optic nerve with high resolution. OCT offers the opportunity to study neurological diseases in an objective and non-invasive manner. Multiple studies and clinical observations support the importance of the OCT in the diagnosis, treatment, and follow-up of optic neuropathies, which has become an important tool in the management of this group of diseases.
